# Fungus-Bacterium Symbionts Promote Plant Health and Performance

**DOI:** 10.1264/jsme2.ME3303rh

**Published:** 2018-09-29

**Authors:** Yong Guo, Kazuhiko Narisawa

**Affiliations:** 1 College of Agriculture, Ibaraki University 3–21–1 Chuuo, Ami, Inashiki, Ibaraki 300–0393 Japan

Anton de Bary (1831–1888) initially presented the term ‘symbiosis’ in 1879 (8), which implies an intimate relationship between organisms of two or more species with a broad spectrum of beneficial, neutral, or harmful relationships. Microbial symbioses are ubiquitous in nature (53); from protists to humans, all plants and animals are inhabited by microbes that comprise the majority of global biodiversity (6). In the past two decades, an increasing number of studies have shown that many plant-associated fungi, including foliar ascomycetes (1), dark septate endophytes (DSEs) (18), arbuscular mycorrhizal fungi (AMF) (4, 47), ectomycorrhizal basidiomycetes (3, 19), and sebacinalean endophytes (40), as well as a few phytopathogenic fungi (27, 31) and neutral fungi living in soil ecosystems (30, 37, 39, 51), harbor phylogenetically diverse bacteria in both epihyphal and endohyphal forms. These bacteria are affiliated to *Alpha-*, *Beta-*, *Gammaproteobacteria*, *Mollicutes*, *Bacillaceae*, *Chitinophaga*, and others, and are known to alter host morphology, sporulation, metabolite production, and even other properties involved in interactions with plants (16, 36, 41, 49). However, symbiotic partners are generally regarded as separate individuals, which has limited the comprehensive assessment of the interaction mechanisms within these holobiont systems (6, 52).

To date, the best-studied fungus-bacterium symbiont system is the phytopathogenic fungus *Rhizopus microsporus* and its endosymbiotic bacterium *Burkholderia rhizoxinica* (31), in which the host is associated with early diverging lineages of terrestrial fungi within Mucoromycotina (5, 42). An interesting multilateral microbial interaction involving *B. rhizoxinica* was shown to cause rice seedling blight (30). *R. microsporus* was considered to be the causative agent of rice seedling blight and the antimitotic agent, rhizoxin, was used to bind β-tubulin in plant tissues as a virulence factor (38, 45). However, it became evident that this virulence factor was produced not by *R. microsporus* itself, but by the endosymbiotic bacterium *B. rhizoxinica* (32, 33). Furthermore, the spore-formatting capacity of *R. microsporus* was completely dependent on the presence of *B. rhizoxinica*, which increased the fitness of both partners for the survival and dispersion of their population (34). This mutually beneficial relationship between a bacterium and fungus has also been reported between the phytopathogenic bacterium *Ralstonia solanacearum*, which contributes to chlamydospore formation in 34 species of fungi across three diverse taxa (Ascomycetes, Basidiomycetes, and Zygomycetes) (43). In this case, *R. solanacearum* produces the secondary metabolite ‘ralsolamycin’ using a non-ribosomal peptide synthetase-polyketide synthase hybrid, which contributes to bacterial invasion into the fungal hyphae and induces chlamydospore formation. Some chlamydospores harbored *R. solanacearum* internally, indicating an endofungal lifestyle for this important plant pathogen. Ecophysiological investigations on fungus-bacterium symbiosis are extremely valuable for suppressing soil diseases caused by microbial pathogens (20, 21, 35, 54).

Coincidentally, fungal-bacterial interactions are also critical to the life cycle development of plant-beneficial fungi. The best known example is the arbuscular mycorrhiza affiliated with Glomeromycotina, as the most widespread component of the plant microbiota that is associated with obligate endosymbiotic bacteria of the rod-shaped Gram-negative ‘*Candidatus* Glomeribacter gigasporarum’ (*Ca*Gg) and the coccoid Gram-positive *Mollicutes*-related endobacteria (MRE) (9, 48). A comparative transcriptomic investigation of the germinating spores of *Ca*Gg-harboring and -cured *Gigaspora margarita* indicated that *Ga*Gg increased fungal sporulation efficiency, thereby enhancing the fungal bioenergetic capacity involved in increasing ATP production and detoxifying reactive oxygen species (36). In turn, *Ca*Gg showed extreme dependence on its host for the supply of nutrients and energy (15). Of note, endobacteria appeared to enhance fungal responsiveness to strigolactones, plant molecules that AMF perceive as branching factors (36). These findings indicated that *Ga*Gg not only improved AMF environmental fitness, but also enhanced symbiotic capacity with specific plants, contributing to their occupation of two distinct niches, in soil and inside root tissues. On the other hand, the fungal hosts of *Dentiscutata heterogama* and *Rhizophagus irregularis* appeared to not only control nutrient and energy supplies to their endobacteria, but also exchanged genetic elements with the endosymbionts of *Ca*Gg and MRE, and even with their plant hosts (25, 48). The genes acquired by *R. irregularis* (*e.g.*, genes encoding MULE transposase, polo kinase, cytotoxin, and HAUS augmin-like protein) may be associated with spore and hyphal growth (25). This horizontal gene transfer may be associated with the evolution and symbiotic adaptation of AMF. Together with the above-described cases, these studies indicated that fungus-bacterium symbioses, including pathogenic and symbiotic lifestyles, play a significant role in plant health.

Besides these important findings, the underlying mechanisms and ecological role of the recently identified *Mortierella*- *Mycoavidus* symbiosis have remained unclear (30, 37). The genus *Mortierella* is also affiliated with early diverging lineages of the terrestrial fungi Mortierellomycotina and is closely related to the phytopathogen *Rhizopus* (42). However, the endobacteria dwelling in the hyphae of this fungal group are more closely related to *Ga*Gg inside AMF rather than *B. rhizoxinica* and *B. endofungorum* inside *R. microsporus* (37). The latter studies reported that *Mycoavidus* and *Ca*Gg appear to have diverged from a common ancestor, whereas *B. rhizoxinica* and *B. endofungorum* may have different ones (5, 41). Previous studies demonstrated that *Mycoavidus* strongly affected host metabolism and reduced growth rates (26, 49). The most recent study revealed that *M. elongata* had the ability to degrade a range of phytotoxic organics in soil and promote plant growth (24). However, limited information is currently available on the ecological role of this fungus-bacterium symbiosis in plant health and performance. Previous studies developed a successful strategy for isolating *Mycoavidus* from the hyphal homogenates of *Mortierella* in accordance with advances in reverse genomics (30), showing that the endobacteria associated with *Mortierella* generally lack key genes for cysteine biosynthesis (14, 41). In the near future, this culture-based approach will assess the functions of the partners of *Mortierella*-*Mycoavidus* symbiosis in plant growth.

Another noteworthy plant-beneficial fungal group is DSEs, intimately associated with symbiotic bacteria (18). DSEs are defined as conidial or sterile ascomycetous fungi that colonize the root tissues of diverse plants intracellularly and intercellularly without any harmful effects or typical mycorrhizal structure formation (2, 23, 29). Previous findings indicated that DSEs have beneficial features for host plants, such as promoting plant growth, enhancing host tolerance against environmental stresses, and deterring encroachment by phytopathogens (10–12, 44). Since DSEs may be easily isolated from the environment and artificially cultured as a granular inoculum for plants (23, 28, 50), they are expected to become useful biofertilizers and bioagents for preventing soil disease.

Until very recently, the DSE *Veronaeopsis simplex* Y34 was considered to harbor the bacterial symbiont, *Agrobacterium pusense* VsBac-Y9, which regulated the carbon metabolism of *V. simplex* Y34 and enhanced the symbiotic relationship with tomato seedlings (17, 18). A series of genes responsible for host interactions with both the fungus and plant were identified in the genome of *A. pusense* VsBac-Y9, such as the genes involved in the secretion systems of type II and type IV, chitinase, siderophore biosynthesis, cobalamin biosynthesis, and acidic polysaccharide succinoglycan biosynthesis (17). Although the interactions between other DSE species and their symbiotic bacteria have not yet been clarified, DSEs generally localize at the center of core microorganisms in heathy plants (46), reflecting an intimate relationship with other microbes. In our field experiment in 2016, an inoculation with *Phialocephala fortinii* LtPE2 significantly increased the abundance of *Pseudomonas* spp. in the rhizosphere of allium and suppressed allium white rot caused by *Sclerotium cepivorum*. This finding also demonstrates that the DSE-bacteria relationship is involved in plant heath.

The benefits of the symbiotic microbiome have been maximized not only by plant-microbe coevolution, but also co-evolution among microbes themselves (13) in an at least 450-million-year history (7, 49). The systematical management of plant-associated microbes may represent the best strategy to achieve a resource-efficient and pathogen-resistant agroecosystem (46). Further polyphasic investigations on molecular-, cultivation-, and bioinformatics-based approaches are needed to understand microbial interactions associated with plants, with a particular focus on fungus-bacterium symbioses.
